# Police as first reponders improve out-of-hospital cardiac arrest survival

**DOI:** 10.1186/s12873-023-00876-w

**Published:** 2023-09-06

**Authors:** Clint Jean Louis, Marta Cildoz, Alfredo Echarri, Carlos Beaumont, Fermin Mallor, Robert Greif, Miguel Baigorri, Diego Reyero

**Affiliations:** 1 Emergency Advanced Ambulance Physician, Prehospital Emergency Services, Navarra Health Services, Avenida Pamplona No.2. 4ª, Barañain, Navarra España 31010 Spain; 2 Regional Coordinator Cardiac-Arrest Code, Citizen Empowerment Program, Navarra Health Services, Pamplona, Navarra Spain; 3 European Resuscitation Council (ERC) Research NET, Brussels, Belgium; 4 Department of Statistics and Operational Research, Public University of Navarra, Pamplona, Navarra Spain; 5 Head of Emergency Transportation Services, Prehospital Emergency Services, Navarra Health Services, Pamplona, Navarra Spain; 6 Emergency Physician, Emergency Department, University Hospital of Navarra, Pamplona, Navarra Spain; 7 Department of Anaesthesiology and Pain Medicine, Bern University Hospital, University of Bern, Bern, Switzerland; 8 School of Medicine, Sigmund Freud University Vienna, Vienna, Austria

**Keywords:** Out-of-hospital cardiac arrest, Police, Defibrillation, First responder, Emergency medical services, Survival, Prehospital

## Abstract

**Background:**

Police forces are abundant circulating and might arrive before the emergency services to Out-of-Hospital-Cardiac-Arrest victims. If properly trained, they can provide basic life support and early defibrillation within minutes, probably increasing the survival of the victims. We evaluated the impact of local police as first responders on the survival rates of out-of-hospital cardiac arrest victims in Navarra, Spain, over 7 years.

**Methods:**

A retrospective analysis of an ongoing Out-of-Hospital Cardiac registry to compare the characteristics and survival of Out-of-Hospital-Cardiac-Arrest victims attended to in first place by local police, other first responders, and emergency ambulance services between 2014 and 2020.

**Results:**

Of 628 cases, 73.7% were men (aged 68.9 ± 15.8), and 26.3% were women (aged 65,0 ± 14,7 years, *p* < 0.01). Overall survival of patients attended to by police in the first place was 17.8%, other first responders 17.4% and emergency services 13.5% with no significant differences (*p* > 0.1). Time to initiating cardiopulmonary resuscitation is significant for survival. When police arrived first and started CPR before the emergency services, they arrived at a mean of 5.4 ± 3 min earlier (SD = 3.10). This early police intervention showed an increase in the probability of survival by 10.1%.

**Conclusions:**

The privileged location and the sole amount of personnel of local police forces trained in life support and their fast delivery of defibrillators as first responders can improve the survival of out-of-hospital cardiac arrest victims.

**Supplementary Information:**

The online version contains supplementary material available at 10.1186/s12873-023-00876-w.

## Background

The annual incidence of out-of-hospital cardiac arrest (OHCA) in Europe is estimated to be between 67 and 170 cases per 100,000 inhabitants [1]. Early cardiopulmonary resuscitation (CPR) and defibrillation improve survival rates [2]. Emergency services cannot easily comply with ideal response times due to fewer resources and wider distances [3–5]. Therefore, an effective strategy in the management of OHCA may benefit from multiple interventions of key agents in the community who may have an advantage in accessing OHCA patients before emergency services [6–8].

It follows that in addition to health care professionals, the police can be dispatched simultaneously when the emergency coordination center receives a call after OHCA. Better knowledge of their municipalities, constant patrolling, and rapid mobility makes it reasonable to consider implicating them in the response system as they arrive before the emergency services on many occasions. Several programs have shown that police trained in Basic Life Support (BLS) reduce the time to CPR and defibrillation and can thus improve the chances of survival [9–15].

Since 2014, OHCA in Navarra, Spain, has been integrated into the Attention to Time-dependent Emergencies Program [16]. That same year, a voluntary BLS training program for police was initiated to integrate them into the Chain of Survival. In 2019, a regional decree made BLS training and the provision of automated external defibrillators (AEDs) for police patrols mandatory [17].

The aim of this study is to evaluate the characteristics of police intervention of OHCA victims in Navarra between 2014 and 2020 and its impact on survival. It aims to fill the knowledge gap regarding implementation and outcomes when police intervention is fully integrated into the dispatch strategy of emergency services in cases of OHCA [18].

## Methods

### Design

We performed a retrospective study of the continuous registry of OHCA in Navarra, part of the national Out-of-Hospital-Cardiac Arrest Registry (OHSCAR), between 2014 and 2020 [19]. The register employs standard Utstein-style definitions, and data are collected by emergency medical services (EMS) personnel. In our case, a thorough revision of the clinical records was also necessary to complete missing data in the OHSCAR-Navarra registry.

The study complies with the Helsinki Declaration and respects the confidentiality of patients and the regulations on the protection of patients’ personal data and was authorized by the Ethics Committee for Clinical Research in Navarra (Ref: PI-2020/60).

### Setting and dispatch system

Navarra is a relatively small region (10,391 km^2^) in Spain with a population of 660.887 inhabitants and has 272 municipalities, of which 47 have municipal police. Over 774 officers serve 420,000 inhabitants in 47 municipalities in the region, 65% of the population. The EMS teams consist of 7 physician- and nurse-staffed advanced life support ambulances, three located in the capital, Pamplona, and the rest in three smaller cities. Non-EMS teams include emergency technicians and firefighters in 51 Basic Life Support ambulance, family medicine physicians and nurses working in 57 primary care zones, mobile and outpatient emergency services in 3 major cities, and rural emergency services located in 42 of the primary care zones. All emergency services offered by these providers are available 24 h a day.

When the emergency coordination center receives a call for a possible OHCA, it activates a protocol consisting of a series of parallel actions, including telephone-assisted CPR, simultaneous dispatch of police and EMS, and other emergency health care services (non-EMS) depending on the location of the OHCA.

First responder is defined as any of the agents (police, EMS, or non-EMS) who arrive first to the OHCA victim.

### Study population

**Fig. 1:**
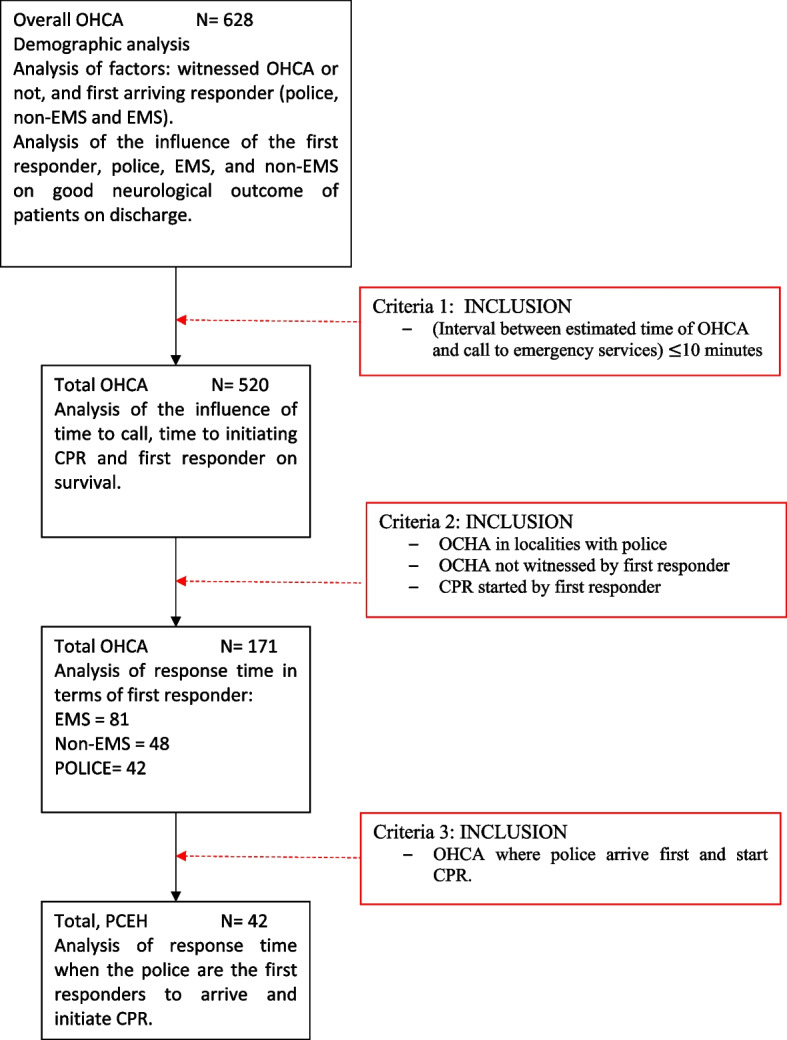
Study population according to inclusion criteria of OHCA cases between 2014 and 2020. [OCHA: out-of-hospital cardiac arrest; CPR: cardiopulmonary resuscitation; EMS: emergency medical services; BLS: Basic Life Support]

The overall study included all OHCA cases in the registry of medical origin, probably cardiac, and excluded cases of traumatic origin, foreign body airway obstruction, drowning, and drug overdose. Those cases with an interval between the estimated time of cardiac arrest and the call to the emergency services of more than 10 min were also excluded, as no survivor was observed after that period. In the remaining cases, we analyzed the influence of various time-related factors on survival: time to alerting the emergency services from the estimated hour of cardiac arrest, time to initiating CPR, and the first responder to arrive at the scene Fig. 1.

For the specific and comparative analysis of response times observed for police, non-EMS and EMS, we included only those victims of OHCA in municipalities with local police, where there was an interval between the moment of cardiac arrest and the moment when CPR was initiated by a first responder, and not by the bystander, and where the cardiac arrest was not witnessed by the first responder. All patients were transferred to the same local reference hospital and the local in-hospital team applied to all patients the same post-resuscitation care.

### Variables and time-related factors

Demographic variables of OHCA victims included age, gender, bystander-witnessed OHCA (yes/no), telephone-assisted CPR (yes/no), location (home, public building, public street or area, nursing home, during ambulance transfer), defibrillation (yes/no), and return of spontaneous circulation (ROSC) and survival at discharge.

*Time to call* is defined as the interval between the estimated hour of OHCA and the hour when the call to the emergency services was made. The estimated time of arrest was assessed by the emergency physician of the EMS at the scene by asking family members or witnessed persons for the time of cardiac arrest as exact as possible to establish the time of cardiac arrest. *Time to CPR* is defined as the interval between the estimated time of OHCA and the start of CPR. *Time to shock* is considered the interval between the estimated time of OHCA and shock application by the first responder.

**Fig. 2:**
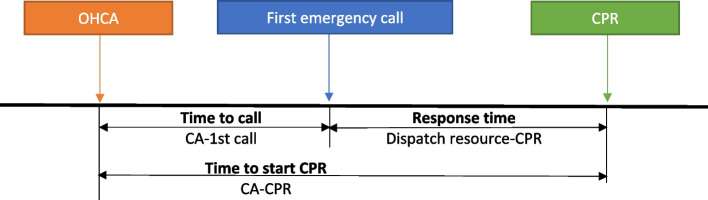
Time intervals during the attention of OHCA. [CA: cardiac arrest; OCHA: out-of-hospital cardiac arrest; CPR: cardiopulmonary resuscitation RCP. AED: automated external defibrillator]

*Response time* refers to the interval between the time of arrival of the first responders and the time of call, which was only recollected by the EMS teams. In the cases of police and non-EMS, this interval was assumed to be the interval between the start of CPR by the first responder and the call to the emergency services (Time to CPR) Fig. 2. All intervals were measured in minutes.

### Outcome

The primary outcomes were ROSC on hospital arrival and survival and neurological status at hospital discharge, employing the *Cerebral Performance Category (CPC*) scale, considering a score of 1 or 2 as good or moderate neurological outcome, respectively [20].

### Statistical analysis

The study compares the characteristics, response times and survival of victims of OHCA attended to in the first place by police, the physician-staffed ambulance EMS and other non-EMS emergency care services. We performed a descriptive analysis for quantitative variables through measures of central tendency and dispersion and for qualitative variables through absolute and relative frequencies. In the case of genre, a Z-test for proportions and for the comparison of age related to genre, we employed a T-Test for two samples.

To compare the mean response times of each first responder (police, non-EMS and EMS) and patients age, we employed analysis of variance (ANOVA) and a Fisher comparison test.

To compare gender, witnessed OHCA, and shockable rhythm/shock proportion across all first responder groups we employed Chi-Square test.

To study survival at discharge with good neurological outcome and ROSC, binary regression logistic models were employed for the variable death (1 = yes, 0 = no) with different groups of independent variables:

- Witnessed OHCA or not
- Age
- Gender
- First responder
- Time to call
- Time to CPR
- Time to CPR and first responder
- Time to CPR and time to call

Good neurological outcome (CPC 1–2) at hospital discharge was evaluated through binary logistic regression with the first responder as an independent variable.

All the tests for goodness of fit performed (Deviance, Pearson, Hosmer–Lemeshow) for the logistic regressions showed a level of significance greater than 0.05, indicating that there was no sufficient evidence to conclude that the model did not conform to the data.

To evaluate the benefit obtained in the cases where police arrived first and initiated CPR, we carried out an adjustment of the probability distribution of the lead time of police before the arrival of the EMS. The headway or lead time is the difference between the arrival of the police and that of the EMS.

To evaluate the increase in the probability of survival with good neurological outcomes due to police integration as first responders in locations with local police, we estimated the probability distribution of the response time when the police arrived first before the other emergency services (EMS and non-EMS). In these cases where the police arrived first, their time of arrival was substituted by the time of arrival recorded by the EMS. For these two probability distributions, we calculated the expected value of the survival function S(t). The increase or gain in the probability of survival obtained due to police intervention is the difference between the above expected values, considering the proportion of times that the police arrived before the other first responders. See Supplementary file 1 for a more detailed account of the mathematical procedure employed.

Data from the registry were transferred to a Microsoft ® Excel 2010 spreadsheet to create a database, from which the statistical analysis was performed through the Minitab ® 19 program.

## Results

### Demography Table 1

**Table 1:** Demographic and OHCA characteristics stratified by first responder (2014 – 2020)

	**Police**		**Non-EMS**		**EMS**		**Total**	
	**N**	%	**N**	%	**N**	%	**N**	%
**Total OHCA**	107	17	196	31.2	325	51.8	628	100.0
Year								
2014	4	5.5	26	35.6	43	58.9	73	100.0
2015	9	16.1	19	33.9	28	50.0	56	100.0
2016	10	14.7	26	38.2	32	47.1	68	100.0
2017	9	11.4	24	30.4	46	58.2	79	100.0
2018	22	22.7	26	26.8	49	50.5	97	100.0
2019	27	19.3	38	27.1	75	53.6	140	100.0
2020	26	22.6	37	32.2	52	45.2	115	100.0
**Gender**								
Male	88	82.2	139	70.9	236	72.6	463	73.7
Female	19	17.8	57	29.1	89	27.4	165	26.3
	*Chi-Square Test p* = *0,082*							
**Age: mean (standrad deviation)**								
Total	61.9 (15.7)		65.7 (15.7)		67.6 (14.2)		66.0 (15.1)	
Male	62.3 (15.1)		64.1 (16.0)		66.6 (13.5)		65.0 (14.7)	
Female	60 (18.3)		69.6 (14.3)		70.3 (15.8)		68.9 (15.8)	
	*Two-way ANOVA p* = *0,001 for gender and p* = *0.005 for first responder*							
**Witnessed OHCA**								
Yes	80	74.8	162	82.7	255	78.5	497	79.1
No	27	25.2	34	17.3	64	19.7	125	19.9
Unknown	-	-	-	-	6	1.8	6	0.9
	*Chi-Square Test p* = 0,262							
**Shockable rhyhtm/shock**								
Yes	36	33.6	58	29.6	108	33.2	202	32.2
No	71	66.4	138	70.4	217	66.8	426	67.8
	*Chi-Square Test p* = 0,645							
**Bystander CPR**								
Yes	32	29.9	54	27.6	130	40.0	216	35.8
Family	17	53.1	23	42.6	82	63.1	122	15.1
Non family bystander	15	46.9	31	57.4	48	36.9	94	20.7
Telephone Assisted	12	37.5	13	24.1	32	24.6	57	9.1
**Location**								
Home	68	63.6	101	51.5	229	70.5	398	63.4
Public street	26	24.3	42	21.4	35	10.8	103	16.4
Work place	2	1.9	5	2.6	9	2.8	16	2.6
Public builiding/área	11	10.3	11	5.6	21	6.5	43	6.9
Health facility/nursing home	0	0.0	30	15.3	8	2.5	38	6.1
During transfer	0	0.0	3	1.5	14	4.3	17	2.7
Other	0	0.0	4	2.0	9	2.8	13	2.1
**ROSC** *p*-value = 0.61								
Yes	46	43.0	77	39.3	141	43.4	264	42.0
No	61	57.0	119	60.7	184	56.6	364	58.0
**Survival at hospital discharge** *p* value = 0.97								
CPC1-2	19	17.8	34	17.4	44	13.5	97	15.5
CPC3-4	0	0.0	2	1.0	3	0.9	5	0.8

A total of 628 cases met the inclusion criteria. Of the 628 cases, 165 (26.3%) were women and 463 (73.7%) were men; there were significantly more men (463, 73.7%) than women victims (*p* < 0.01), with mean ages of 68.9 (± 15.8) and 65 (± 14.7) years, respectively, which were also significantly different (*p* < 0.01).

The police arrived in first place in 107 (17%), the EMS in 325 (51.8%) and the non-EMS in 196 (31.2%) cases. In all cases, the police initiated BLS and delivered at least one shock in 36 (33.64%) of the cases, while the non-EMS in 58 (29.6%) and the EMS in 108 (33.3%) of the cases did not show significant differences (*p* < 0.1). There were no significant differences in AED application (*p* < 0,1). The majority of the OHCA events occurred at home (63.4%) and were witnessed in 79.1% of the cases, and bystander CPR started in 35.8% of them. Most of the cases attended in the first place by police were at home (63.6%) and in public areas (24.3%). The non-EMS responders arrive first in most OHCA cases occurring in nursing homes (15.3% of their cases, which represents 78.9% of the cases in this location).

The proportion of patient gender across groups is significant at the $\alpha$ level of 0.1 (*p* = 0.082); EMS proportion men = 72.6%; 95% CI (0.67; 0.77); non-EMS proportion men = 82.2%; 95% CI (0.74; 0.90); police proportion men = 71%; 95% CI (0.64;0.77).

Age across groups is significantly different even when considering gender as confounder (two-way ANOVA *p* = 0.001 for gender and *p* = 0.005 for first responder); EMS age mean = 67.6; 95% CI (66.0; 69.3); non-EMS proportion men = 65.7; 95% CI (59.1; 64.7) police proportion men = 61.9; 95% CI (66.0; 69.3).

Witnessed cardiac arrest and shockable rhythm across groups are not significant at the $\alpha$ level of 0.1.

### The influence of witnessed events and first responders on survival and on ROSC (*n* = 628)

Overall survival with good CPC (CPC 1–2) was 15.5%. There was no statistically significant difference between the three first responder types (*p* > 0.1): in cases attended to by police teams in the first place, survival was (17.8%), those attended to by non-EMS (17,4%), and those by EMS teams (13.5%).

Witnessing an OHCA significantly influenced survival [OR = 3.98; 95%CI (1.80,8.82); *p* < 0.01]. Whether the OHCA was witnessed or not, the type of first responder did not significantly influence survival (OR Police/Non-EMS = 0.96; 95% CI (0.52, 1.77); OR EMS/Non-EMS = 0.75; 95% CI (0.47, 1.21); OR EMS/ Police = 0.78, 95% CI (0.44, 1.40); *p*- = 0.45).

Both age and gender showed significant differences with respect to survival, [OR = 0.96; 95%CI (0.95; 0.98); *p* < 0.001] and [OR Masc-Fem = 2.53; 95%CI (1.4;4.6); *p* < 0.05] respectively.

Witnessing an OHCA significantly influenced ROSC [OR = 3.32; 95%CI (2.09;5.25); *p* < 0.001]. Whether the OHCA was witnessed or not, the type of first responder did not significantly influence survival [OR Police/Non-EMS = 1.17; 95%CI (0.72;1.88); OR EMS/Non-EMS = 1.18; 95%CI (0.83;1.70); OR EMS/ Police = 1.02; 95%CI (0.65;1.58); p- = 0.64]. Age significantly influenced survival [OR = 0.99; 95%CI (0.98;0.99); *p* < 0.05] and gender no [OR Masc-Fem = 1.16; 95%CI (0.81;1.67); *p* = 0.42].

### Influence of time to call, CPR and first responder on survival and on ROSC (*n* = 520)

The time to call significantly influenced survival [OR = 0.88; 95% CI (0.78;0.99), *p* < 0.05] and ROSC [OR = 0.90; 95% CI (0.83;0.98), *p* < 0.05].

Time to initiating CPR significantly affected ROSC [OR = 0.96; 95% CI (0.83;0.98), *p* < 0.05] and survival [OR = 0.9153; 95% CI (0.8663, 0.9611), *p* < 0.01]. There is a reduction in the probability of both ROSC and survival of nearly 10% for every minute of delay.

Mean time intervals of each first responder are shown in Table 2.

**Table 2:** Time intervals for first responders attending OHCA victims

	**Police** Mean (SD) CI 95%	**EMS** Mean (SD) CI 95%	**Non – EMS** Mean (SD) CI 95%	*p*-value
**N**	42	74	48	
**Time to call (min)**	2.19 (2.67) (1.48; 2.90)	1.593 (1.99) (1.08; 2,10)	2.542 (2.54) (1.877; 3.21)	0.072
**Time to CPR (min)**	6.62(3.69) (5.14; 8.10)	11.00 (5.29) (9.89; 12.12)	4.50 (5.05) (3.12; 5.88)	< 0.001
**Mean response time (min)**	4.48 (3.59) (3.16; 5.80)	9.51 (4.56) (8.52; 10.51)	3.04 (4.54) (1.81; 4.28)	< 0.001

The complete survival model with all confounder factors (first responder, patient’s age and gender, and time to initiating CPR) maintains the significance of time to initiating CPR [OR = 0.91; 95%CI (0.86;0.96);*p*-value < 0.01], gender [OR = 2.60; 95%CI (1.36;4.97); *p*-value < 0.01], and age [OR = 0.96; 95%CI (0.94; 0.97); *p*-value < 0.001], but shows no significance to the type of first responder [OR Police/Non EMS = 1.04; 95% CI (0.52;2.01); OR EMS/Non-EMS = 0.96; 95% CI (0.57;1.61); OR EMS/ Police = 0.93, 95% CI (0.48;1.78); *p*-value = 0.97].

The complete ROSC model with all confounder factors (first responder, patient’s age and gender, and time to initiating CPR) maintains the significance of time to initiating CPR [OR = 0.95); 95%CI (0.92;0,98);*p*-value < 0.01], and age [OR = 0,90; 95%CI (0.97;0,99);*p*-value < 0.01], but shows no significance at the $\alpha$ level of 0.05 to the type of first responder [OR Police/Non EMS = 1.52; 95%CI (0.89;2.62); OR EMS/Non EMS = 1.61; 95%CI (1.08;2.40); OR EMS/ Police = 1.06; 95%CI (0.634;1.77); *p*-value > 0.05], and gender [OR = 0.99; 95%CI (0.97;0.99); *p*-value = 0.61].

As there were no differences between type of first responder on survival, but a difference in survival depending on the response time, we investigated further to determine if there was a significant contribution or benefit on the probability of survival, whenever the police arrived first and started CPR.

The probability function of survival related to the time to initiating CPR (minutes) is the following:

$$S\left(t\right)=\frac{{e}^{(-\mathrm{1,035}-\mathrm{0,0898}t)}}{(1+{e}^{\left(-\mathrm{1,035}-\mathrm{0,0898}t\right)})}$$

### Study of response time depending on first responder to OHCA (*n* = 171)

The mean response times of the first responders were significantly different (*p*-value < 0.001). The mean times (standard deviation) are non-EMS 3.0 (4.53) 95% CI (1.8; 4.28), police 4.5 (3.6) 95% CI (3.16; 5,80), EMS 9.5 (4.6) 95% CI (8.5; 10.51).

**Fig. 3:**
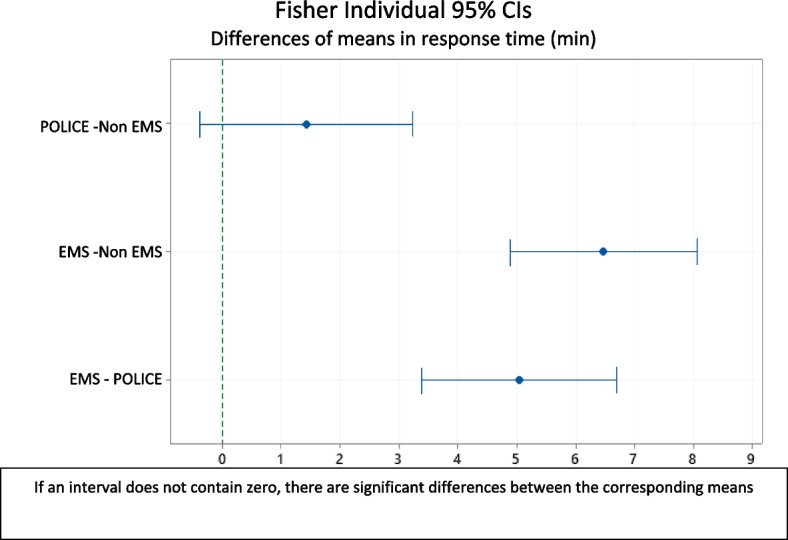
Comparison of mean response times among first responders (police, non-EMS and EMS)

Fisher pairwise comparisons show that the time to arrival of the EMS is significantly higher than that of non-EMS and the police (Fig. 3).

**Fig. 4:**
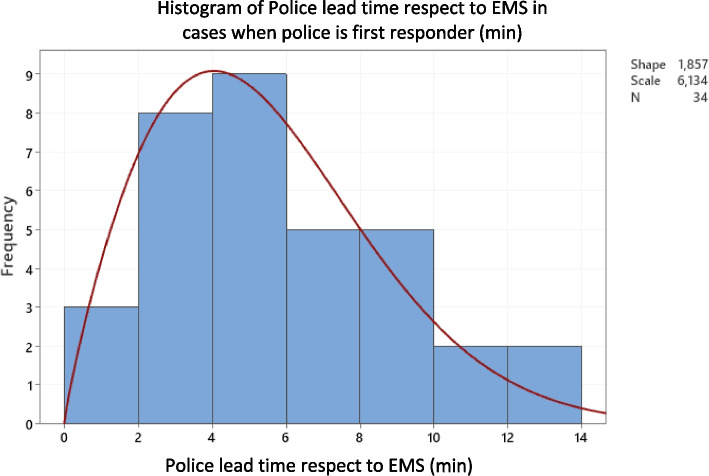
Police lead time to arrival at OHCA victims compared to EMS

In 42 of the 171 cases (24,6%), the police were the first responders and initiated CPR, and the EMS arrived at a mean time of 5.4 min later (SD = 3.1). The police lead time is adjusted to a Weibull distribution with a shape of 1.857 and a scale of 6.134. (Fig. 4).

The time to CPR when the police, the non-EMS and EMS intervene follows a Weibull distribution parametrized to scale 10.03 and shape 1.78, while the time to CPR when only the EMS and non-EMS intervene follows a Weibull distribution parametrized to scale 11.51 and shape 2.061.

The expected value of the survival function when police intervene is 14.1%, while the expected value of the survival function when only the other first responders (Non-EMS and EMS) intervene is 12.8%. This indicates a 10.1% increase or gain in the probability of survival due to faster response times shown by police intervention.

## Discussion

This study evaluated the impact of municipal police as the first responders to arrive at OHCA victims compared to non-EMS and EMS services. We observed that survival depended on when the first responder arrived, independent of who it was, police, non-EMS, or EMS. The trained police obtained similar survival outcomes compared to other emergency care professionals.

Further analysis of cases where police arrived first and started CPR (24.6%) showed that they arrived significantly earlier than the EMS teams, with a mean difference of 5.4 ± 3.1 min. This meant a 10% increase in the probability of survival thanks solely to the early police intervention.

These results strengthen the strategic decision to fully integrate the police in the emergency response to OHCA.

In Spain, although there is evidence on training programs and the availability of AEDs we did not find any similar study on police intervention and survival after OHCA [21, 22].

There are obstacles to the implementation of police training programs, including police reluctance to attend OHCA victims attributed to a lack of confidence or clear directives in applying the AED, conflict with other police tasks, delays in departure and concerns over legal issues [23, 24]. However, after the initial pilot and voluntary program in 2014, we observed that as reports emerged on police successfully resuscitating OHCA victims, the program rapidly spread to other towns with municipal police, overcoming the abovementioned obstacles and evolving into a legally established part of police training. Nevertheless, we believe that despite biannual mandatory recertification, regular training sessions within their internal formation, the availability of training material, debriefing sessions after attending OHCA events, and medical and psychological support are necessary to maintain their motivation and improve their competence and confidence in applying high-quality BLS [14].

The mean response times of non-EMS teams were also significantly better than those of EMS teams. A far greater number and variety of the defined non-EMS teams, their distribution throughout the region and specific dispatch motives can explain the better response times in this study.

Independent of which first responder arrived first, the shorter the interval between cardiac arrest and calling the emergency services (time to call), the higher the percentage of patients who survived [25, 26]. Nearly 80% of the OHCA events were witnessed, and in 35.8% of the cases, bystander CPR was initiated.

Hence, it is important to empower citizens with BLS education combined with the implementation of public access defibrillation (PAD) programs [27]. Unlike the police and other first responders, such as firemen or lifeguards, the legislation in Navarra permits any citizen to use an AED with no mandatory certification [17]. This has enormous advantages because through BLS education and public AED access, it is much easier to plan for and train citizens as adults and even before they complete secondary school.

In localities with no municipal police, and especially in rural areas, community-based citizen first-responder programs combined with smartphone activation linked to the emergency coordination center and judiciously distributed public access AEDs gain more relevance, while non-EMS and EMS teams make way [27, 28].

Our study has some limitations. The information from the Navarra OHCA registry is introduced by the EMS teams who attend the victim and do not include OHCA cases where EMS teams have not intervened. This may create a selection bias, which we consider small as the EMS teams are dispatched in the majority of the OHCA events, making it largely representative of the population. Nevertheless, non-EMS teams should participate in the registry.

The evaluation of first responder intervention on survival did not include the influence of hospital care but centered on time intervals to arrival. However, as all patients in our region were treated in the same hospital by the same in-hospital team under the same local post-resuscitation treatment and care, we assume all patients were treated in the same way, which might diminish the possibility of such a bias. Future investigations should account for the influence of in hospital treatment of cardiac arrest victims.

In recent years, there has been an improvement in data recollection, but it remains incomplete; hence, consultation of the patient’s clinical records was necessary. Other authors have described the same limitation, attributing it to greater consumption of time, dependence on a diversity of individuals, and the demands of clinical practice [1, 29].

The police response time was estimated based on the time to call and the start of CPR. It would be reasonable for police to provide data to the registry while maintaining patient confidentiality to obtain more precise information on response times and on their performance before the emergency personnel arrive. Cities and municipalities differ in size, population density and availability of police officers, which evidently influence an early emergency response to OHCA victims.

## Conclusion

The intervention of police trained in BLS and provided with AEDs shows similar survival rates of OHCA victims compared to emergency services and independent of the reduction in response times. Additionally, the potentially faster response times make their integration into the immediate emergency response to OHCA an effective and complementary strategy to improve survival.

### Supplementary Information

**Additional file 1.**

## Data Availability

The datasets used and/or analysed during the current study are available from the corresponding author on reasonable request. The data generated or analysed and the formulae employed during this study are included in this published article [and its supplementary information files].

## Abbreviations

AEDs: Automated external defibrillators
BLS: Basic life support
CPC: Cerebral performance category
CPR: Cardiopulmonary resuscitation
EMS: Emergency medical services
OHCA: Out-of-hospital cardiac arrest
OHSCAR: Out-of-hospital cardiac arrest registry
ROSC: Return of spontaneous circulation
