# Supplementary material for: Police as first reponders improve out-of-hospital cardiac arrest survival
Source: BMC Emerg Med. 2023 Sep 6;23:102. doi: 10.1186/s12873-023-00876-w (PMC10481462; doi:10.1186/s12873-023-00876-w)
Supplement: Supplementary file 1 — Additional file 1. [file 12873_2023_876_MOESM1_ESM.docx]

**APPENDIX 1. METHODOLOGY TO STUDY INCREASE IN SURVIVAL DUE TO EARLY POLICE INTERVENTION**

Let $S(t)$ be the probability function of survival related to the time to initiating CPR. Let $F_{SP}(t)$ and $f_{SP}(t)$) be the probability distribution function and the density function of the time to CPR, respectively, when the police, EMS and Non-EMS intervene. Both, $F_{SP}(t)$ and $f_{SP}(t)$), are estimated by using the current data base.

Let also denote by $F_{NP}(t)$ and $f_{NP}(t)$ the probability distribution function and the density function of the time to CPR, respectively, when only EMS and Non-EMS intervene. The estimation of these functions are done by substituting the registered time to CPR by the interval between OCHA and time of arrival registered by the EMS teams in the cases where the police are the first responders to arrive. Observe that the difference between both intervals, the registered one and its substitute, is the temporal gain obtained by police intervention after they are simultaneously dispatched by the emergency coordination center.

The expected probability of survival when an OHCA occurs in each of the probability distributions of time to CPR is:

$$E\left( S_{SP}\left( t \right) \right)=\int_{0}^{\infty} S\left( t \right) f_{SP}\left( t \right) dt$$

$$E\left( S_{NP}\left( t \right) \right)=\int_{0}^{\infty} S\left( t \right) f_{NP}\left( t \right) dt$$

The increase in survival attributed to police intervention is obtained from the difference:

$$\Delta S=E\left( S_{SP}\left( t \right) \right)-E\left( S_{NP}\left( t \right) \right)$$
